# The Triglyceride–Glucose Index, a Marker of Insulin Resistance, Is Associated with the Myocardial Performance Index in Asymptomatic Subjects

**DOI:** 10.3390/medicina61060987

**Published:** 2025-05-27

**Authors:** Necip Nas, Muzaffer Aslan, Semih Saglik, Hafize Uzun

**Affiliations:** 1 Department of Internal Medicine, Medical Faculty, Siirt University, 56100 Siirt, Turkey; 2 Department of Cardiology, Medical Faculty, Siirt University, 56100 Siirt, Turkey; mzfraslan56@hotmail.com; 3 Department of Radiology, Siirt Training and Research Hospital, 56000 Siirt, Turkey; drsmhsglk@gmail.com; 4 Department of Medical Biochemistry, Faculty of Medicine, Istanbul Atlas University, 34403 Istanbul, Turkey; huzun59@hotmail.com

**Keywords:** subclinical left heart failure, metabolic syndrome, asymptomatic subjects, myocardial performance index, triglyceride–glucose index

## Abstract

*Background and Objectives*: The myocardial performance index (MPI) is a diagnostic tool that assesses both the systolic and diastolic function of ventricles. The MPI provides a comprehensive view of the overall efficiency of the heart’s pumping ability, making it a valuable tool for detecting early signs of heart dysfunction, even in the absence of overt symptoms. In this regard, we aimed to explore the relationship between the triglyceride–glucose (TyG) index and subclinical heart failure (HF), as well as its correlation with the MPI, in asymptomatic patients visiting a routine cardiology outpatient clinic. The study specifically excluded individuals with known diabetes, hypertension, and HF, focusing instead on those who had undergone 12 h fasting blood glucose (FBG) and triglyceride (TG) tests. *Materials and Methods*: The study included 125 patients with FBG, TG, total cholesterol (TC), low-density lipoprotein cholesterol (LDL-C), and high-density lipoprotein cholesterol (HDL-C) data after the exclusion criteria were applied. *Results*: When asymptomatic patients were categorized as MPI normal or MPI (+) subjects, significant differences were found between the groups in terms of body mass index (BMI), metabolic syndrome (MetS) components, and serum TG values. Pearson correlation analysis revealed a significant and positive correlation between the MPI and TyG index (r = 0.358, *p* < 0.001). Regression analysis was used to determine the effective parameters in subclinical left ventricular dysfunction (SCLVD). In univariate regression analysis, obesity, the presence of MetS, serum TG, and the TyG index were identified as risk factors. In multivariate regression analysis, the TyG index was found to be the independent risk factor. *Conclusions*: The positive association found between the MPI and TyG index suggests a link with metabolic disorders and myocardial performance. Obesity, the presence of MetS, serum TG, and the TyG index were identified as risk factors for SCLVD in asymptomatic patients. Notably, the TyG index was identified as an independent risk factor for SCLVD, highlighting its potential role in the early identification and risk stratification of individuals at risk for cardiac dysfunction. These findings suggest that monitoring the TyG index could provide valuable insights into subclinical heart dysfunction, particularly in patients with metabolic abnormalities.

## 1. Introduction

The myocardial performance index (MPI), also known as the TEI-Doppler index, can be defined as a non-invasive method for assessing the systolic and diastolic function of the left ventricle (LV). The MPI is an indicator of both ventricular systolic and diastolic function. It is calculated by dividing the sum of the isovolumetric contraction time and isovolumetric relaxation time by the LV ejection time [1]. The MPI, determined by Doppler waveforms, has many advantages. It can be widely used in cardiac evaluation, is easily obtainable and repeatable, is independent of ventricular cavity geometry, and is not affected by blood pressure [2]. However, a disadvantage is that it is influenced by preload [3].

The risk of atherosclerotic cardiovascular diseases (CVDs), such as myocardial infarction (MI), strokes, peripheral vascular diseases, insulin resistance (IR), and type 2 diabetes mellitus (T2DM), is believed to stem from a shared underlying pathogenesis. These conditions contribute to the development of CVDs and are collectively referred to as metabolic syndrome (MetS) [4]. IR is a condition where the body’s cells do not respond effectively to insulin, a hormone that helps to regulate blood glucose levels. This means that the body needs to produce more insulin to keep its blood glucose levels in check, leading to elevated insulin levels (hyperinsulinemia). Over time, this can increase the risk of T2DM, heart disease, and other complications.

IR has been recognized not only as a pathogenic cause, but also as a predictor of CVD both in general populations and in people with diabetes. Therefore, the development of useful and reliable screening tools to detect IR and predict cardiovascular risks is particularly important. The triglyceride–glucose index (TyG index) is a useful marker for IR, which can help to predict the likelihood of developing T2DM and other metabolic disorders. The TyG index is a simple and effective method for estimating IR without the need for complex or invasive tests like oral glucose tolerance tests (OGTTs) or insulin sensitivity testing. MetS and IR are key contributors to cardiovascular risk, and they can lead to an increased MPI, which indicates suboptimal heart function [5]. The MPI offers a non-invasive way to assess early myocardial dysfunction, helping to detect subtle heart abnormalities that may not yet be clinically apparent, but could lead to more serious cardiovascular events in the future. The TyG index and MPI are useful tools for early intervention, allowing for personalized treatment strategies to reduce the risk of complications such as heart disease and T2DM. Monitoring these indices can help to detect issues before they manifest clinically, improving long-term health outcomes.

The MPI is a reproducible marker of global ventricular function and has been associated with adverse cardiovascular outcomes, including heart failure and mortality [1,2,3]. Although not yet widely used in routine practice, its ability to detect subclinical myocardial dysfunction has garnered increasing interest. When combined with metabolic markers such as the TyG index, the MPI may enhance cardiovascular risk stratification.

The aim of this study is to investigate the relationship between the TyG index and subclinical heart failure (HF), as well as its association with the MPI in asymptomatic patients attending routine cardiology outpatient clinic visits after excluding those with known diabetes, hypertension (HT), or HF, focusing on those who have undergone 12 h fasting blood glucose and TG tests.

## 2. Materials and Method

### 2.1. Ethical Approval

All procedures performed in this study involving human participants were in accordance with the ethical standards of the institutional and/or national research committee and with the Helsinki declaration and its later amendments or comparable ethical standards. Approval for this study was obtained from Siirt University, Ethics Committee for Clinical Research (27 December 2024; Decision no: 125949; Number: 2024/12/01/09).

### 2.2. Study Populations

**Figure 1:**
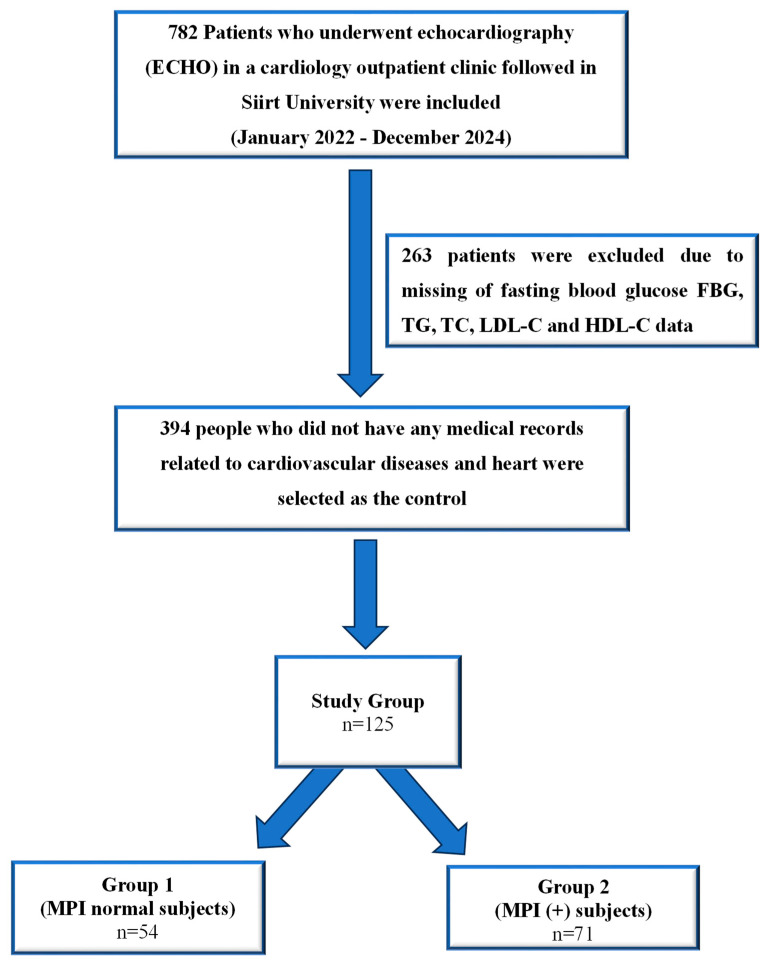
A flowchart of the selection of cases. FBG: fasting blood glucose, TG: triglyceride, TC: total cholesterol, LDL-C: low-density lipoprotein cholesterol, HDL-C: high-density lipoprotein cholesterol.

The medical records of 782 patients who underwent echocardiography (ECHO) at a cardiology outpatient clinic between January 2022 and December 2024 were retrospectively evaluated. All patients included in this study were of Turkish ethnicity. After applying the exclusion criteria, 125 patients were included, because 263 patients were missing fasting blood glucose (FBG), TG, total cholesterol (TC), low-density lipoprotein cholesterol (LDL-C), and high-density lipoprotein cholesterol (HDL-C) data (Figure 1). Patients attending routine cardiology outpatient clinic visits were screened retrospectively from their files.

Detailed cardiovascular history and physical examination information were obtained from the medical records of the patients. Twelve-lead electrocardiograms (ECGs) of all patients were obtained. In addition, all patients were screened in detail for hyperlipidemia, active smoking, CVD, and family history as risk factors.

### 2.3. The Inclusion Criteria

After excluding patients with asymptomatic and known T2DM, HT, and HF, patients with 12 h fasting blood glucose and lipid parameters were included. Individuals with no cardiovascular events were included.

### 2.4. The Exclusion Criteria

Patients with T2DM, HF (left ventricular ejection fraction (LVEF) < 50%), hypertrophic obstructive cardiomyopathy, moderate to severe valvular disease, previous coronary artery bypass or valve surgery, renal failure, severe hepatic failure, previous stroke, recent MI, atrial fibrillation, endocarditis, active infection, malignancy, and those with unclear images were excluded. Previously known T2DM, HT and medications, if any, were questioned and these patients were excluded from the study.

Body mass index (BMI) was calculated using the formula weight (kg)/[height (m)]^2^.

The patients in this study were categorized based on their BMI using the WHO classification system, as follows: 18.5–24.99 kg/m^2^ for normal weight, 25.0–29.99 kg/m^2^ for overweight, 30.0–34.99 kg/m^2^ for class I obesity, 35.0–39.99 kg/m^2^ for class II obesity, and ≥40 kg/m^2^ for class III obesity [6].

Hemogram, lipid, and biochemical parameters determined in blood samples obtained by the peripheral venous route in the morning after 12 h of fasting were obtained from the patients’ records.

### 2.5. Definition of Metabolic Syndrome (MetS)

Metabolic syndrome was defined according to the criteria established by the National Cholesterol Education Program Adult Treatment Panel III (NCEP ATP III), which are commonly used in clinical practice. A diagnosis of MetS was made when an individual met at least three of the following five components [7].

1. Central obesity, defined as a waist circumference of ≥ 94 cm in men and ≥80 cm in women
2. Elevated serum triglycerides, defined as TG levels of ≥150 mg/dL
3. Reduced HDL, defined as HDL < 40 mg/dL in men and <50 mg/dL in women
4. Hypertension, defined as either a systolic blood pressure of ≥130 mmHg and/or a diastolic blood pressure of ≥85 mmHg or a history of hypertension
5. IR, defined as either a history of diabetes or the use of antidiabetic drug treatment

### 2.6. Blood Pressure Measurement

After 20 min of rest, blood pressure was measured twice intermittently with an Omron blood pressure monitor in a sitting position, and the average of the two measurements was taken. Limit values were determined according to the NCEP ATP III diagnostic criteria, and the results are expressed as “mm Hg” [7].

### 2.7. Electrocardiogram (ECG) Scan

Retrospective analysis was conducted on 12-lead ECG recordings obtained using the AT-102 device (Schiller AG, Baar, Switzerland). The ECGs were recorded at a paper speed of 25 mm/sec with a calibration of 10 mV/cm.

### 2.8. Cardiac Ultrasound Examinations

**Figure 2:**
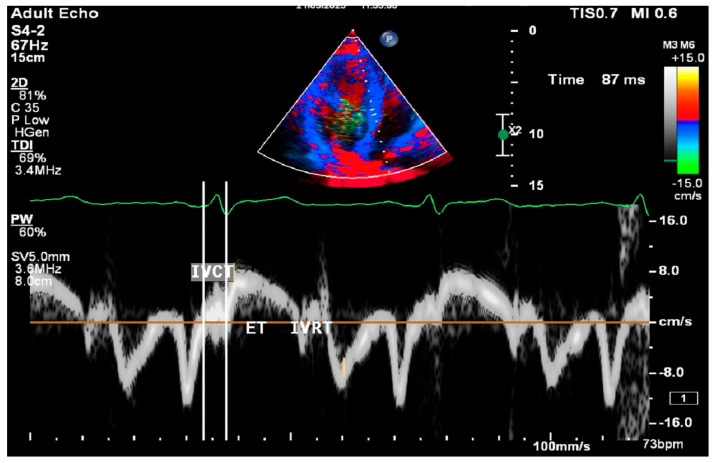
Test image in conventional M-mode, B-mode, and Doppler parameters.

All procedures were performed using Philips Epiq 7 ultrasound systems (Philips Ultrasound; Bothel, WA, USA) and a 2.5 MHz FPA probe. Conventional M-mode, B-mode, and Doppler parameters were measured according to the American Society of Echocardiography guidelines [8]. Left ventricular end-diastolic diameter (LVDd), left ventricular end-systolic diameter (LVDs), posterior wall (PW), and interventricular septum (IVS) thicknesses were measured. Left ventricular ejection fraction (LVEF) was measured from the apical four-chamber view using the Modified Simpson method. Mitral E, A waves, and E wave deceleration time were obtained by PW Doppler placed at the tip of the mitral leaflets in the apical four-chamber window. E/A ratios were calculated for each patient. Measurements were made by placing tissue Doppler on the septal and lateral edges of the mitral valve in the apical four-chamber window. Early diastolic peak (Em), late diastolic peak (Am), and peak systolic flow (Sm) velocities were measured. Isovolumetric relaxation time (IVRT), isovolumetric contraction time (IVCT), and ejection time (ET) were also found (Figure 2).

The myocardial performance index (MPI) was calculated using the data obtained from Doppler echocardiography and the following formula [9]. Patients with an MPI of 0.5 and above were defined as having subclinical left ventricular dysfunction (SCLVD) [10]. The left ventricular outflow tract (LVOT) flow was assessed by pulse wave Doppler in the apical five-chamber window immediately adjacent to the aortic valve.

In all patients, the presence of a presystolic wave was assessed just before LVOT flow.

$$LVOT\;flow=\frac{Isovolumic\;Relaxation\;Time\;(IVRT)+Isovolumic\;Contraction\;Time\;(IVCT)}{Ejection\;Time\;(ET)}$$

### 2.9. Statistical Analysis

The data analysis of our study was performed using SPSS 20.0 software (Statistical Package for the Social Sciences, Chicago, IL, USA). Variables related to qualitative data are expressed as number (*n*) and percentage (%), and variables related to quantitative data are expressed as mean ± standard deviation (SD). In the evaluation of the study data, the Student’s t test was used for intergroup comparisons of normally distributed variables and the Mann–Whitney U test was used for intergroup comparisons of non-normally distributed parameters. The Chi-square test or Fisher’s exact test were used to compare categorical variables depending on the sample size. Univariate and multivariate binary logistic regression analyses were used to determine the risk factors affecting subclinical left ventricular dysfunction. The relationship between the parameters was analyzed with Pearson correlation coefficient. Linear regression was used to examine the relationship between the TyG index and MPI as the dependent variable. In the study cases, a receiver operating characteristic (ROC) curve analysis was used to investigate the performance of important criteria in determining disease diagnosis. The significance level for statistical results was accepted as *p* < 0.05.

## 3. Results

Baseline characteristics and comparisons of variables between groups are summarized in Table 1. A total of 125 patients were included in the study, and 47 were female and 78 were male. The average age of all patients was 45.5 ± 10 years, while it was 44.7 ± 9.9 years for males and 47 ± 10.1 years for females. MPI normal subjects were categorized as group 1 and MPI (+) subjects as group 2. There were statistically significant differences in BMI, MetS characteristics, and serum TG values between both groups (*p* < 0.05 for all). There were no statistically significant differences in both systolic and diastolic blood pressures between the two groups. The TyG index of group 2 was significantly higher than that of group 1 (*p* < 0.001).

Regression analysis was used to determine the effective parameters in SCLVD. In the univariate regression analysis, obesity (OR, 2.34; ≥30, versus < 30; 95% CI, 1.08–5.05), the presence of MetS (OR, 3.46; yes, versus no; 95% CI, 1.64–7. 28), serum TG (OR, 1.01; per mg/dL increment; 95% CI, 1.004–1.018), and TyG index (OR, 1.042; per unit increment; 95% CI, 0.348–3.12) were identified as risk factors. In the multivariate regression analysis, the TyG index (OR, 0.14 per unit increment; 95% CI, 0.035–0.57) was found to be the independent risk factor (Table 2).

**Figure 3:**
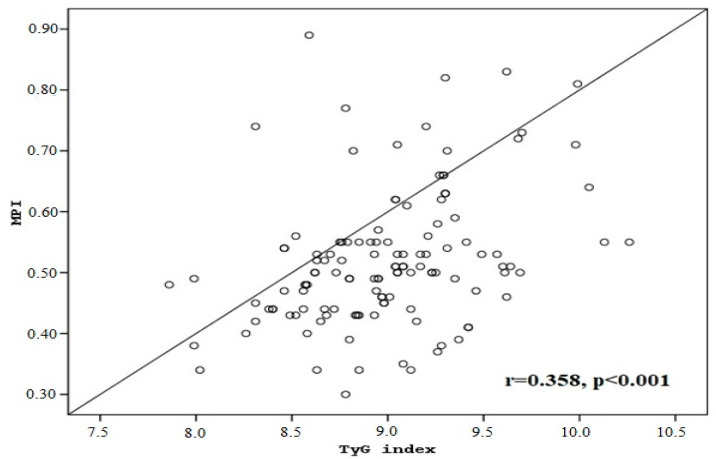
Correlation between triglyceride–glucose (TyG) index and myocardial performance index (MPI) in all groups.

Pearson correlation analysis revealed a significant and positive correlation between the MPI and TyG index (r = 0.358; *p* < 0.001) (Figure 3).

**Figure 4:**
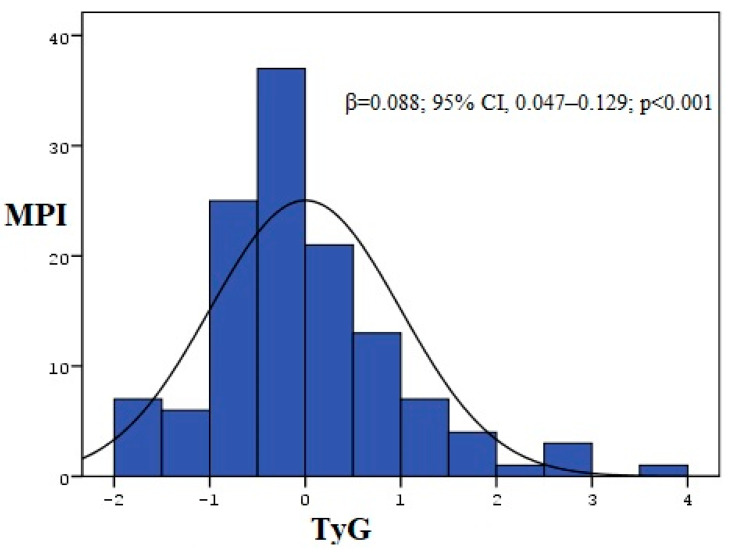
Gaussian graph of the relationship between MPI and TyG index. TyG index, triglyceride–glucose index; MPI, myocardial performance index.

We used a Gaussian graph to visually represent the distribution pattern of the continuous variables—the TyG index and MPI—following the linear regression analysis. This type of graph helps to illustrate whether the data follow a normal distribution and to better understand the overall trend and variability in the relationship between these two indices. It also provides a more comprehensive visualization of the underlying data structure, supporting the assumptions of linear regression and offering clearer insight into the nature of the association. In addition, in linear regression analysis, a significant and positive correlation was found between the MPI and TyG index (β = 0.088; 95% CI, 0.047–0.129; *p* < 0.001) (Figure 4).

In the linear regression analysis, a significant and positive correlation was found between the MPI and TyG index. In a comparison of the TyG index values between both groups, statistically, MPI (+) patients had a higher TyG index than MPI (−) patients.

Horizontal lines within each box represent mean values, and the lower and upper lines of each box represent the minimum and maximum values, respectively. Vertical lines and whiskers indicate 95% CIs.

Regression analysis was used to determine the effective parameters in SCLVD. In the univariate regression analysis, obesity (OR, 2.34; ≥30, versus < 30; 95% CI, 1.08–5.05), the presence of MetS (OR, 3.46; yes, versus no; 95% CI, 1.64–7. 28), serum TG (OR, 1.01; per mg/dL increment; 95% CI, 1.004–1.018), and TyG index (OR, 1.042; per unit increment; 95% CI, 1.035–1.051) were identified as risk factors. In the multivariate regression analysis, the TyG index (OR, 1.038 per unit increment; 95% CI, 1.031–1.046) was found to be the independent risk factor (Table 2).

## 4. Discussion

The most important finding of the present study was that BMI, characteristics of MetS, and serum TG values were significantly different between the two groups, suggesting that these factors may be associated with the presence of SCLVD. Additionally, the TyG index was significantly higher in MPI-positive patients, further supporting the potential role of the TyG index as an important marker in identifying individuals at a higher risk for cardiac dysfunction. This suggests that higher TyG values may be linked to increased cardiovascular risk, particularly in individuals with an abnormal MPI. In asymptomatic patients, identifying SCLVD as early as possible is critical in predicting patients at a high risk of developing HF. The American Society of Echocardiography also recommends the use of Doppler in routine cardiac evaluation [11]. The MPI can be defined as a non-invasive method that measures LV systolic and diastolic function. The MPI is an indicator of ventricular systolic and diastolic function [1]. In a study conducted by Moller et al. [12] on healthy individuals, it was found that the normal MPI value was 0.34 ± 0.04, and this value increased in patients who had experienced an MI. In another study, the MPI value was 0.39 ± 0.05 in healthy individuals, while in patients with dilated cardiomyopathy, the value was found to be 0.59 ± 0.10 [13]. Spencer et al. [14] investigated the relationship between age and the MPI. They evaluated individuals with no history of vascular disease, normal sinus rhythm, normal blood pressure, no significant valvular heart disease, normal wall motion, normal LV mass, and normal systolic function. As a result, they found statistically significant changes in the MPI, IVRT, and ET. The significant linear correlation between age and the MPI was attributed to the increase in IVRT observed between the ages of 16 and 80. At the same time, it has been reported that elevated MPI values in elderly men are an important determinant in the development of congestive HF (CHF) in the following years [15]. In our study, no significant correlation was found between age and the MPI. This may be due to the small sample size and the lack of long-term follow-up. The MPI has also been reported to be a useful parameter in the follow-up of patients with HF.

In the present study, the significant differences observed between the MPI normal and MPI-positive groups in terms of BMI, components of MetS, and serum triglyceride (TG) values suggest that these factors may be associated with subclinical myocardial dysfunction, even in asymptomatic individuals. This highlights the potential utility of the MPI as a diagnostic tool in identifying early cardiac dysfunction in patients who may not yet exhibit overt symptoms. The findings also emphasize the importance of monitoring metabolic risk factors, such as BMI and serum TG levels, in assessing cardiovascular health, particularly in individuals who are otherwise asymptomatic. All patients included in this study were of Turkish ethnicity. As the study was conducted at a single center in Turkey, there was no ethnic or racial variation among the participants. Therefore, potential differences in cardiovascular disease risk due to ethnic background were not applicable in this cohort. In a study comparing patients with T2DM without a history of HT or coronary or valvular disease and control samples, it was observed that the MPI increased significantly in the patient group compared to the control group [16]. In another study conducted in diabetic patients without significant heart failure and CVD, a strong correlation was found between the MPI and the degree of albuminuria. The researchers suggested that the MPI may be a sensitive marker for the diagnosis of LV dysfunction in patients with T2DM [17]. Kılıç et al. [18] detected higher MPI values and SCLVD in patients with MetS compared to the normal population, underscoring the increased cardiovascular risk associated with MetS. MetS is known to be a cluster of risk factors, including obesity, IR, dyslipidemia, and HT, all of which can contribute to myocardial dysfunction over time. The association of *p*-wave signal width (PSW) positivity with SCLVD in this patient group further suggests that electrical and structural changes in the heart, potentially linked to metabolic disturbances, may precede or coincide with the development of subclinical cardiac dysfunction. This highlights the importance of the early identification and monitoring of MetS in preventing or managing latent cardiac issues. Kumar et al. [19] reported that MetS is a strong predictor of subclinical myocardial dysfunction in individuals without clinically apparent heart disease, highlighting the silent but significant impact of MetS on cardiac health. Even in the absence of overt symptoms or diagnosed heart disease, the components of MetS can contribute to early myocardial changes that may not be detectable through routine clinical assessments. This finding suggests that MetS may serve as an early indicator of cardiac dysfunction, emphasizing the importance of proactive monitoring and intervention in individuals with MetS to prevent the progression to clinically apparent heart disease [19,20]. This reinforces the importance of addressing MetS comprehensively, even in individuals with normal blood pressure, to prevent future cardiovascular complications.

In the present study, the finding that the TyG index was higher in the MPI (+) group compared to the MPI normal group suggests a potential link between metabolic disturbances and subclinical myocardial dysfunction. There is also a significant and positive correlation between the MPI and TyG index. The TyG index, which reflects IR and metabolic imbalance, may serve as an early marker for individuals at risk of developing cardiac dysfunction, even in the absence of overt symptoms. This reinforces the notion that elevated TyG levels could be indicative of early myocardial impairment, highlighting their potential role in identifying asymptomatic individuals at a higher risk for heart-related issues before clinical manifestations appear. The TyG index might be an independent predictor of CVD severity and cardiovascular outcomes in non-ST-segment elevation acute coronary syndrome (NSTE-ACS). Higher TyG values are associated with a poorer myocardial performance [21]. The positive relationship between the MPI and TyG highlights the potential of the TyG index as a useful tool for assessing cardiovascular risk, particularly in individuals with metabolic abnormalities.

In the present study, the TyG index emerged as the only independent predictor of SCLVD in the multivariate analysis, highlighting its potential as a sensitive marker of early myocardial impairment, independent of traditional risk factors such as obesity and MetS. This finding aligns with previous research by Yang et al. [22], which identified the TyG index as a strong predictor of acute kidney injury and poor renal outcomes in critically ill HF patients, emphasizing its broader role in cardiorenal metabolic risk. Additionally, elevated TyG levels have been associated with increased mortality and rehospitalization rates in HFpEF patients, further supporting its prognostic value in heart failure management [23,24,25,26]. These findings suggest that the TyG index may serve as a practical and cost-effective tool for early cardiovascular risk stratification and outcome prediction [27,28,29,30,31].

The MPI, TyG index, and MetS play crucial roles in understanding cardiovascular risk, particularly in individuals without overt symptoms. The higher the MPI, the greater the likelihood of subclinical or early myocardial dysfunction, which is crucial for risk stratification in patients at risk of HF. MetS is a significant predictor of subclinical myocardial dysfunction in individuals without clinically evident heart disease. MetS is a strong predictor of subclinical myocardial dysfunction in subjects free of clinically apparent heart disease. The TyG index could be used as an effective and non-invasive marker to guide early detection, risk stratification, and tailored treatment plans in HF patients, especially those with MetS or IR. Its predictive value for both cardiovascular outcomes highlight its broad clinical utility in HF management. In asymptomatic patients, elevated MPI and TyG indices can reveal underlying cardiac dysfunction and metabolic issues, such as IR or components of MetS, before clinical symptoms manifest. This underscores the importance of monitoring these markers in individuals without obvious heart disease to detect subclinical cardiac dysfunction early. Oksen et al. [32] demonstrated that increased oxidative stress in diabetic patients was associated with higher MPI values (≥0.5), indicating early SCLVD and supporting the MPI as a reliable marker for detecting asymptomatic myocardial impairment. In a study by Akyüz et al. [33], SCLVD was defined as an MPI of ≥0.5 in patients with preserved ejection fraction, supporting the use of the MPI as a sensitive marker for early ventricular dysfunction in asymptomatic hypertensive patients. Our results support the results of Oksen et al. [32] and Akyüz et al. [33]. Detecting SCLVD early through tools like the MPI and TyG can lead to more proactive management, potentially preventing the progression of symptomatic HF.

### 4.1. Novelty and Clinical Implications

The novelty of this study lies in its focus on asymptomatic individuals without known cardiovascular disease, evaluating the association between the TyG index—a readily accessible marker of insulin resistance—and subclinical myocardial dysfunction, as measured by the MPI. While previous research has examined the TyG index in populations with established cardiovascular conditions, limited data exist regarding its relationship with early, asymptomatic cardiac impairment. Our findings demonstrate a significant correlation between elevated TyG levels and an impaired MPI, suggesting that metabolic disturbances may contribute to cardiac dysfunction even before clinical symptoms appear. This study adds to the growing body of evidence supporting the TyG index as a valuable, non-invasive tool for early cardiovascular risk assessment and highlights its potential role in preventive strategies aimed at identifying high-risk individuals before overt disease develops.

### 4.2. Limitations of the Study

This study has several limitations that should be acknowledged. First, it was conducted retrospectively, which may introduce selection bias and limit the ability to control for potential confounding factors. Second, the sample size was relatively small and derived from a single center, which may limit the generalizability of the findings. Third, the cross-sectional nature of the study prevents us from establishing causal relationships between the TyG index, MPI, and subclinical SCLVD. Fourth, long-term clinical outcomes were not assessed, which limits the ability to determine the prognostic implications of the observed associations. Fifth, there was an absence of insulin level measurements, which prevented direct evaluation of the relationship between insulin resistance and the TyG index.

In conclusion, the integration of the MPI, TyG index, MetS, and their relationship with SCLVD provides a more comprehensive understanding of early cardiovascular risk, particularly in asymptomatic individuals. The early identification of these markers allows for timely intervention and management, potentially preventing the progression to overt HF. While the current findings highlight significant associations, the need for larger prospective studies is critical for confirming causality and understanding the broader implications for clinical practice in preventing and managing CVDs.

## Figures and Tables

**Table 1:** Baseline characteristics and comparison of variables among groups.

	MPI			
Parameters	Group 1 (*n* = 54)	Group 2 (*n* = 71)	Total (*n* = 125)	*p* Values
Age (years)	45.8 ± 9.5	45.3 ± 10.4	45.5 ± 10	0.788 ^a^
Female	46 ± 10.1	47.8 ± 10.3	47 ± 10.1	0.550 ^a^
Male	45.7 ± 9.4	43.9 ± 10.3	44.7 ± 9.9	0.431 ^a^
Gender *n* (%)				0.795 ^c^
Female	21 (38.9%)	26 (36.6%)	47 (37.6%)	
Male	33 (61.1%)	45 (63.4%)	78 (62.4%)	
BMI (kg/m^2^)	27.8 ± 5.1	30.1 ± 5.6	29.1 ± 5.5	0.028 ^a^
Obesity *n* (%)				0.039 ^b^
<30 kg/m^2^	40 (74.1%)	39 (54.9%)	79 (63.2%)	
≥30 kg/m^2^	14 (25.9%)	32 (45.1%)	46 (36.8%)	
Smoking *n* (%)				0.062 ^c^
No	39 (72.2%)	39 (54.9%)	78 (62.4%)	
Yes	15 (27.8%)	32 (45.1%)	47 (37.6%)	
Metformin *n* (%)				0.627 ^c^
No	39 (72.2%)	54 (76.1%)	93 (74.4%)	
Yes	15 (27.8%)	17 (23.9%)	32 (25.6%)	
Metabolic Syndrome *n* (%)				0.001 ^b^
No	36 (66.7%)	26 (36.6%)	62 (49.6%)	
Yes	18 (33.3%)	45 (63.4%)	63 (50.4%)	
SBP (mmHg)	131.3 ± 16.4	133.3 ± 14.7	132.4 ± 15.4	0.494 ^d^
DBP (mmHg)	84.4 ± 8.3	83.5 ± 9.5	83.9 ± 9.1	0.575 ^d^
Glucose (mg/dL)	101.9 ± 15.6	108 ± 20.1	105.3 ± 18.4	0.069 ^d^
Triglyceride (mg/dL)	148.1 ± 53.5	189 ± 72.7	171.3 ± 68	0.001 ^d^
TyG index	8.77 ± 0.39	9.14 ± 0.41	8.98 ± 0.44	<0.001 ^d^

^a^ Student’s *t*-test with mean ± standard deviation (SD). ^b^ Fisher’s Exact test with *n* (%). ^c^ Chi-Square with *n* (%).^d^ Mann–Whitney U-test with median ± interquartile range (IQR). Abbreviations: MPI, myocardial performance index; BMI, Body Mass Index; TyG index, triglyceride–glucose index; SBP; systolic blood pressure, DBP; diastolic blood pressure.

**Table 2:** Univariate and multivariate binary logistic regression analyses for identifying risk factors associated with subclinical left ventricular dysfunction (SCLVD).

	Univariate		Multivariate	
	*p* Values	OR (CI 95%)	*p* Values	OR (CI 95%)
Obesity (kg/m^2^)				
≥30, against <30	0.03	2.34 (1.08–5.05)	ns	
Metabolic Syndrome				
Yes, against no	0.001	3.46 (1.64–7.28)	ns	
Triglyceride (mg/dL)				
per mg/dL increment	0.002	1.01 (1.004–1.018)	ns	
TyG index				
per unit increment	<0.001	1.042 (1.035–1.051)	0.006	1.038 (1.031–1.046)

NS, not significant; OR, Odds ratio; CI, Confidence interval; TyG, triglyceride–glucose.

## Data Availability

The data that support the findings of this study are available on request from the corresponding author. The data are not publicly available due to privacy or ethical restrictions.

## References

[1] Artola Arita V., Beigrezaei S., Franco O.H. (2024). Risk factors for cardiovascular disease: The known unknown. Eur. J. Prev. Cardiol..

[2] GBD 2019 Diseases and Injuries Collaborators (2020). Global burden of 369 diseases and injuries in 204 countries and territories, 1990–2019: A systematic analysis for the Global Burden of Disease Study 2019. Lancet.

[3] Vaduganathan M., Mensah G.A., Turco J.V., Fuster V., Roth G.A. (2022). The global burden of cardiovascular diseases and risk: A compass for future health. J. Am. Coll. Cardiol..

[4] Yusuf S., Joseph P., Rangarajan S., Islam S., Mente A., Hystad P., Brauer M., Kutty V.R., Gupta R., Wielgosz A. (2020). Modifiable risk factors, cardiovascular disease, and mortality in 155 722 individuals from 21 high-income, middle-income, and low-income countries (PURE): A prospective cohort study. Lancet.

[5] Tei C., Ling L.H., Hodge D.O., Bailey K.R., Oh J.K., Rodeheffer R.J., Tajik A.J., Seward J.B. (1995). New index of combined systolic and diastolic myocardial performance: A simple and reproducible measure of cardiac function—A study in normals and dilated cardiomyopathy. J. Cardiol..

[6] World Health Organization (2020). WHO Guidelines on Physical Activity and Sedentary Behaviour.

[7] Expert Panel on Detection, Evaluation, and Treatment of High Blood Cholesterol in Adults (2001). Executive Summary of The Third Report of The National Cholesterol Education Program (NCEP) Expert Panel on Detection Evaluation And Treatment of High Blood Cholesterol In Adults (Adult Treatment Panel III). JAMA.

[8] Lang R.M., Badano L.P., Mor-Avi V., Afilalo J., Armstrong A., Ernande L., Flachskampf F.A., Foster E., Goldstein S.A., Kuznetsova T. (2015). Recommendations for cardiac chamber quantification by echocardiography in adults: An update from the American Society of Echocardiography and the European Association of Cardiovascular Imaging. Eur. Heart J. Cardiovasc. Imaging.

[9] Harjai K.J., Scott L., Vivekananthan K., Nunez E., Edupuganti R. (2002). The Tei index: A new prognostic index for patients with symptomatic heart failure. J. Am. Soc. Echocardiogr..

[10] Cheung M.M., Smallhorn J.F., Redington A.N., Vogel M. (2004). The effects of changes in loading conditions and modulation of inotropic state on the myocardial performance index: Comparison with conductance catheter measurements. Eur. Heart J..

[11] Nagueh S.F., Smiseth O.A., Appleton C.P., Byrd B.F., Dokainish H., Edvardsen T., Flachskampf F.A., Gillebert T.C., Klein A.L., Lancellotti P. (2016). Recommendations for the Evaluation of Left Ventricular Diastolic Function by Echocardiography: An Update from the American Society of Echocardiography and the European Association of Cardiovascular Imaging. J. Am. Soc. Echocardiogr..

[12] Moller J.E., Sondergaard E., Poulsen S.H., Egstrup K. (2001). The Doppler echocardiographic myocardial performance index predicts left ventricular dilatation and cardiac death after myocardial infarction. Cardiyology.

[13] Takasaki K., Otsuji Y., Yoshifuku S., Kuwahara E., Yuasa T., El Rahim A.E.R.A., Matsukida K., Kumanohoso T., Toyonaga K., Kisanuki A. (2004). Noninvasive estimation of impaired hemodynamics for patients with acute myocardial infarction by Tei index. J. Am. Soc. Echocardiogr..

[14] Spencer K.T., Kirkpatrick J.N., Mor-Avi V., Decara J.M., Lang R.M. (2004). Age dependency of the Tei index of myocardial performance. J. Am. Soc. Echocardiogr..

[15] Arnlöv J., Ingelsson E., Risérus U., Andrén B., Lind L. (2004). Myocardial performance index, a Doppler-derived index of global left ventricular function, predicts congestive heart failure in elderly men. Eur. Heart J..

[16] Karvounis H.I., Papadopoulos C.E., Zaglavara T.A., Nouskas I.G., Gemitzis K.D., Parharidis G.E., Louridas G.E. (2004). Evidence of left ventricular dysfunction in asymptomatic elderly patients with non-insulin-dependent diabetes mellitus. Angiology.

[17] Orem C., Küçükosmanoğlu M., Hacihasanoğlu A., Yilmaz R., Kasap H., Erdoğan T., Kaplan Ş., Çelik Ş. (2004). Association of Doppler-derived myocardial performance index with albuminuria in patients with diabetes. J. Am. Soc. Echocardiogr..

[18] Kılıç R., Aslan M., Nas N., Güzel T. (2023). Relationship between presystolic wave and subclinical left ventricular dysfunction as assessed by myocardial performance index in patients with metabolic syndrome. Int. J. Cardiovasc. Imaging.

[19] Voulgari C., Moyssakis I., Papazafiropoulou A., Perrea D., Kyriaki D., Katsilambros N., Tentolouris N. (2010). The impact of metabolic syndrome on left ventricular myocardial performance. Diabetes Metab. Res. Rev..

[20] Sreenivasa Kumar M.L., Rajasekhar D., Vanajakshamma V., Latheef K. (2014). Impact of metabolic syndrome on global left ventricular function: As evaluated by the myocardial performance index. J. Saudi Heart Assoc..

[21] Mao Q., Zhou D., Li Y., Wang Y., Xu S.C., Zhao X.H. (2019). The Triglyceride-Glucose Index Predicts Coronary Artery Disease Severity and Cardiovascular Outcomes in Patients with Non-ST-Segment Elevation Acute Coronary Syndrome. Dis. Markers.

[22] Yang Z., Gong H., Kan F., Ji N. (2023). Association between the triglyceride glucose (TyG) index and the risk of acute kidney injury in critically ill patients with heart failure: Analysis of the MIMIC-IV database. Cardiovasc. Diabetol..

[23] Khalaji A., Behnoush A.H., Khanmohammadi S., Mardasi K.G., Sharifkashani S., Sahebkar A., Vinciguerra C., Cannavo A. (2023). Triglyceride-glucose index and heart failure: A systematic review and meta-analysis. Cardiovasc. Diabetol..

[24] Zhang F., Hou X. (2024). Association between the triglyceride glucose index and heart failure: NHANES 2007–2018. Front. Endocrinol..

[25] Li X., Chan J.S.K., Guan B., Peng S., Wu X., Lu X., Zhou J., Hui J.M.H., Lee Y.H.A., Satti D.I. (2022). Triglyceride-glucose index and the risk of heart failure: Evidence from two large cohorts and a mendelian randomization analysis. Cardiovasc. Diabetol..

[26] Zheng H., Chen G., Wu K., Wu W., Huang Z., Wang X., Chen Z., Cai Z., Cai Z., Lan Y. (2023). Relationship between cumulative exposure to triglyceride-glucose index and heart failure: A prospective cohort study. Cardiovasc. Diabetol..

[27] Zhou Q., Yang J., Tang H., Guo Z., Dong W., Wang Y., Meng X., Zhang K., Wang W., Shao C. (2023). High triglyceride-glucose (TyG) index is associated with poor prognosis of heart failure with preserved ejection fraction. Cardiovasc. Diabetol..

[28] Huang R., Wang Z., Chen J., Bao X., Xu N., Guo S., Gu R., Wang W., Wei Z., Wang L. (2022). Prognostic value of triglyceride glucose (TyG) index in patients with acute decompensated heart failure. Cardiovasc. Diabetol..

[29] Yang S., Shi X., Liu W., Wang Z., Li R., Xu X., Wang C., Li L., Wang R., Xu T. (2023). Association between triglyceride glucose-body mass index and heart failure in subjects with diabetes mellitus or prediabetes mellitus: A cross-sectional study. Front. Endocrinol..

[30] Fang Y., Shen J., Lyu L. (2024). Value of the triglyceride-glucose index and related parameters in heart failure patients. Front. Cardiovasc. Med..

[31] Huang R., Lin Y., Ye X., Zhong X., Xie P., Li M., Zhuang X., Liao X. (2022). Triglyceride-glucose index in the development of heart failure and left ventricular dysfunction: Analysis of the ARIC study. Eur. J. Prev. Cardiol..

[32] Oksen D., Aslan M. (2024). Impact of oxidative stress on myocardial performance in patients with diabetes: A focus on subclinical left ventricular dysfunction. BMJ Open Diabetes Res. Care.

[33] Akyüz A.R., Turan T., Gürbak İ., Korkmaz L., Ağaç M.T., Çelik Ş. (2016). The relationship between presystolic wave and subclinical left ventricular dysfunction in asymptomatic hypertensive patients. Blood Press. Monit..

